# Farmer Mental Health Interventions: A Systematic Review

**DOI:** 10.3390/ijerph19010244

**Published:** 2021-12-26

**Authors:** Terasa Younker, Heidi Liss Radunovich

**Affiliations:** Department of Family, Youth and Community Sciences, University of Florida, Gainesville, FL 32611, USA; hliss@ufl.edu

**Keywords:** farmers’ mental health, farmer suicide, farming stress, mental health interventions, systematic review

## Abstract

The prevalence of mental health disorders and suicide amongst agricultural producers is a global problem. Community leaders, researchers, policymakers, and clinicians have mobilized to develop programs to address this issue. This study reviewed a wide range of mental health interventions targeting farmer mental health spanning over 50 years and examined their reported effectiveness and constraints. A total of ninety-two articles on farmer mental health were included in a final systematic review. Most articles were written concerning mental health literacy and peer and paraprofessional support interventions in the United States and Australia. Among the 56 studies reporting empirical evaluative data, 21 were mixed-method, 20 quantitative, 11 qualitative, and 5 literature synthesis. Non-experimental, self-reported, and qualitative data suggest efficacy of mental health literacy programs, peer and paraprofessional support, and community-based and agroecological interventions. However, most interventions were not subject to rigorous evaluation and only one intervention was evaluated using a control condition. The heterogeneity of existing studies and paucity of rigorous evaluation proscribes firm conclusions related to program-type efficacy. This review demonstrates that there is still a need for a stronger and broader evidence base in the field of farmer mental health interventions, which should focus on both holistic, multi-component programs and targeted approaches.

## 1. Introduction

Since the 1980s, a wealth of literature has documented mental health issues among different populations of agricultural producers across the geographical, political, cultural, and socioeconomic spectrum [1]. Even during the best of times, agricultural work—defined here as cultivation, domestication, horticulture, arboriculture, and vegeculture, as well as forms of livestock management including aquaculture—exposes producers to uncontrollable and unpredictable conditions such as fickle weather patterns, unpredictable animals and machinery, role conflict, time pressure, long hours of physically demanding and repetitive labor, and social isolation [2]. Acute and chronic stressors which overwhelm an individual’s coping capacity can lead to heightened levels of psychological distress, mental health issues, and suicide [3]. However, the psychosocial stress experienced by primary producers has been tremendously exacerbated by rapid, radical changes in global food systems [4]. What was once a relatively self-sufficient family-farm-based model of agriculture has been transformed into a technology and market-oriented global “industry”, which extends from agricultural production, to sophisticated agriscience, and agribusiness. These changes have forced many farmers out of the profession and forced those who remain to grapple with greater levels of uncertainty regarding what will happen to their livelihoods [5].

Although existing research is insufficient to provide anything near a comprehensive portrait, studies from around the world have documented significantly higher rates of depression, anxiety, and suicide among farmers than the general population [6,7,8,9,10,11,12,13,14,15,16]. In the United States, male agricultural managers die by suicide at nearly twice the rate of men in the general population [17], and recent studies in the Midwest have found that ⅔ of producers reported anxiety disorders, and over half reported depression [16]. In the UK, 88% of farmers under the age of 40 rank poor mental health as their greatest challenge today [18].

It should be noted that high rates of mental health issues have also been documented globally among farmworker populations in high-income countries [19]. However, as the sets of issues affecting farmworkers, who are often migratory, undocumented, low-paid, and perform the most difficult and dangerous agricultural labor, are distinct from those of primary producer farmers, they are excluded from this review.

Farmers have also been more likely to report other symptoms of psychological distress including burnout [20], hopelessness [21], and loss of self-esteem [22]. Risk factors for mental health issues and suicide among farmers have been identified as financial distress [11,16,23,24], pesticide exposure [25,26,27,28], social isolation [23,29,30], health issues and chronic pain [2,31,32,33,34,35], poor access to healthcare [23,29,32,36,37], access to lethal means [34,38,39,40], weather uncertainty [41,42], regulation compliance and paperwork [20,29,43], and a sense of marginalization and beleaguerment [44,45,46].

Recent evidence suggests that the mental health and suicide crises faced by farming communities are growing increasingly dire as globalization, trade liberalization and financial deregulation, resource scarcity, rural depopulation, and climate change accelerate [23,47,48,49,50,51]. Work-related stress and distress are also highly associated with suicide among agricultural producers: the supply-chain disruptions caused by the COVID-19 pandemic brought this into sharp relief with stories of desperate agricultural producers dying by suicide as their unsold produce was dumped into ditches and plowed into the ground [52].

**Figure 1:**
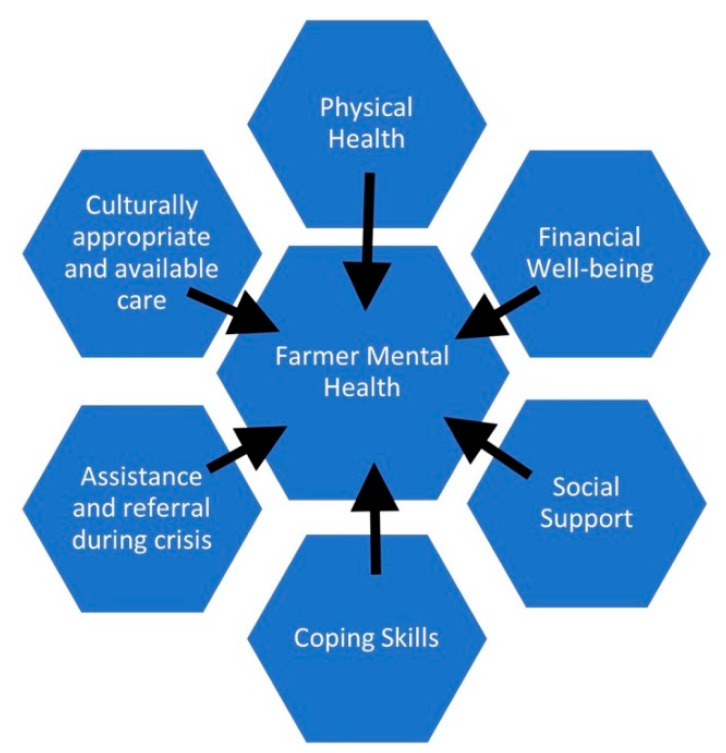
Drivers of farmer mental health.

Although the drivers of mental wellness are well established [11,12,13,14,15,29,36,37,40] (see Figure 1), providing relief to farmers is fraught with challenges. The cost of mental healthcare is prohibitive for many farming families, and even when money is not an issue, access is limited by a severe global shortage of mental health providers [53]. This shortage is exacerbated by maldistribution and unequal access to mental healthcare, which disproportionately affects low-income countries and rural areas, home to the majority of the world’s farmers [53,54]. Widespread stigma toward mental illness and deeply held cultural values of stoicism, hard work, and self-reliance further limit access to traditional forms of mental healthcare [55,56,57].

Part of the tragedy of these crises is that the persistent agricultural mental health issues manifested by this population are highly preventable or treatable [37,58]. Since at least the 1970s, communities across the globe have responded to the increases in farmer mental health and suicide crises by developing and implementing a wide variety of programs, policies, and other interventions [59,60,61]. The available evidence suggests that some of these interventions have been effective, while others have made little impact. In this article, we review approximately 90 interventions, characterized broadly into four categories, developed to improve mental health outcomes or reduce suicide rates among agricultural producers and in agricultural communities. We also examined their reported effectiveness and constraints (if any) to serve as a reference for researchers, policymakers, and clinicians as they mobilize to develop programs for their own communities.

To date there has been no systematic literature review of mental health interventions—defined here as programs and policies aiming to reduce psychological distress, promote overall mental wellness, improve specific mental health outcomes and reduce suicide—targeting agricultural producers. In 2019, Hagen and colleagues published a scoping review of mental health outcomes and interventions among farming populations worldwide, but this review did not include studies published before 1998, and did not specifically examine intervention outcomes [62]. It also included programs targeting farmworkers whose issues and needs may vary from producers. Furthermore, in 2019, Inwood and colleagues published the results of an online scan of resources to present a baseline typology of current mental health programs and response efforts to farmer distress in the 12-state Extension North Central Region. However, these results are limited and do not include evaluative data [63].

## 2. Materials and Methods

This systematic literature review, with no protocol registration, was based on the JBI recommendations [64] and reported according to the Preferred Reporting Items for Systematic Reviews and Meta-Analyses (PRISMA; [65]) protocol. It is intended to answer the question, “What interventions intended to improve the mental health of farmers have been reported in the academic literature, and what is the evidence on their effectiveness and limitations?” The Population-Intervention-Comparison-Outcome-Study Design (PICOS) approach, a modification of the PICO framework, was applied to systematically define the eligibility criteria (Appendix A). The PICOS strategy was guided by the World Health Organization definitions of mental health, mental illness, mental health promotion and protection, and mental health care and treatment [66].

### 2.1. Selection Procedures

The primary author conducted a comprehensive search of peer-reviewed academic articles from May to August 2021 in five electronic databases: PsycINFO, PubMed, Scopus, Agricola, and Google Scholar. The following keywords were utilized in the search: mental health, behavioral health, depression, anxiety, stress, distress, stressors, crisis, suicide, suicidal, psychological, wellbeing, well-being, burnout, and resilience, in combination with farmer, farming, agricultural, and producer, in combination with intervention, evaluation, social support, emotional support, psychological support, program, promotion, prevention, and help.

We initially conducted the search in the academic databases PsycNET, PubMed, Agricola, and Web of Science. However, these searches resulted in only 21 articles eligible for inclusion, all published after 2000. Our work in Extension has made us aware of interventions conducted during the 1980s Farm Crisis, and we determined that it was important to capture this literature as well. We expanded our search to the crawler-based Google Scholar to capture this literature. Because Google Scholar includes grey and other non-academic literature and purports to display results in order of relevance, we only screened the first 1000 results (out of 2,500,000). The full electronic database search generated 4133 English language articles (excluding duplicates). The title screening yielded 1354 articles, which were then screened by abstract using the following criteria:

1. Does the study address issues of mental health or known risk factors of mental health?
2. Are farming populations the target of such interventions?

**Figure 2:**
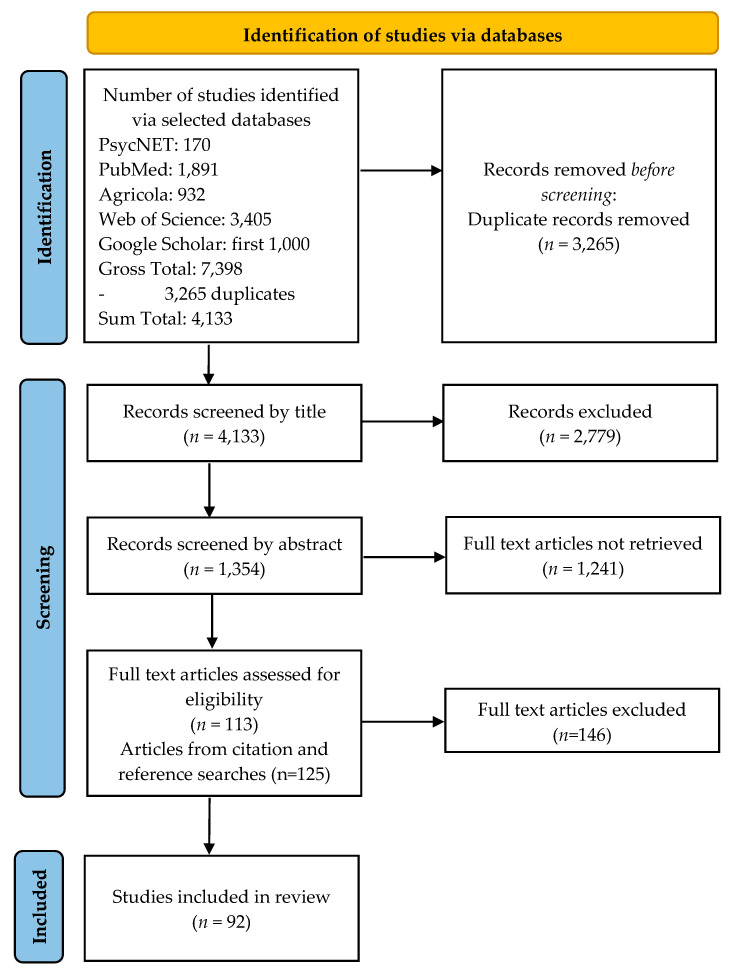
Schematic for identifying studies.

If an article appeared to meet both the above criteria in the abstract screening (“yes” to questions 1. and 2.), the full text was retrieved for review with the full criteria (see Appendix A for details). If the article did not appear to meet the above criteria, it was excluded from further review. A total of one hundred and thirteen full text articles were assessed according to the full criteria, and 1241 articles were excluded at the abstract stage. Citation and reference searches yielded an additional 125 articles, which were reviewed in full. A total of ninety-two articles met full inclusion criteria after full-text review (see Appendix B for a detailed table of included articles). A total of one hundred and forty-six articles were excluded at the full text screening stage. Given that our goal was to summarize interventions within this field so that practitioners and researchers can better understand what has been tried, and that a large proportion of studies published on this topic did not report evaluative data, we chose not to use study quality as a criterion for inclusion. Figure 2 provides the roadmap followed for the studies selection. The secondary author oversaw all aspects of the selection procedures. There was full consensus between the authors regarding study inclusion and exclusion.

Among the articles excluded during the abstract screening process, 437 were analyses of risk factors to farmer mental health, 349 were unrelated to farmer mental health, 246 addressed farmer mental health but did not include information on interventions, 117 addressed mental health interventions that did not primarily target farming populations, 46 addressed mental health interventions in “agricultural regions” without explicitly targeting primary producers, 33 addressed suicide means restriction, and 18 were not published in peer-reviewed academic journals.

### 2.2. Data Collection Process

Search results were imported into Endnote Reference Manager (Clarivate. Philadelphia, PA, USA) to check for duplicates. The primary author extracted data from eligible articles using Microsoft Excel (Microsoft Corporation. Redmond, WA, USA) and NVivo (QSR International. Melbourne, VIC, Australia), a qualitative data analysis and literature review software. Extracted data included study design, aims, type of intervention, delivery method, geographical area, targeted population, methodology, measurement methods for evaluating efficacy, and outcomes.

## 3. Systematic Review Results

### 3.1. Study Characteristics

**Figure 3:**
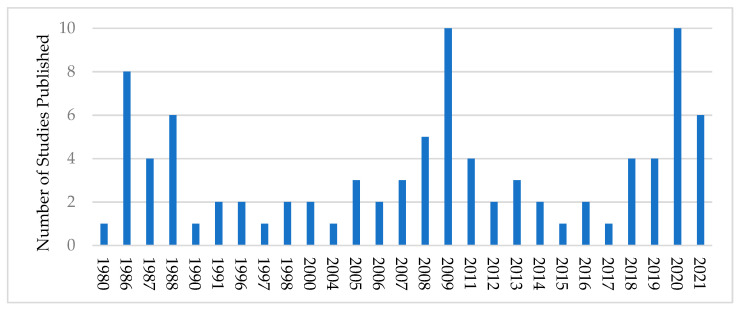
Number of farmer mental health intervention studies from 1980 to August 2021.

The earliest article identified in the search was published in 1980, the most recent in August 2021. Most studies addressed a single intervention and reported some evaluative data (n = 48), followed by studies addressing single interventions with descriptions only (n = 21), studies examining multiple interventions with descriptions only (n = 13), studies addressing multiple interventions reporting evaluative data (n = 6), and recommendations for intervention programming (n = 4). Most articles were written concerning interventions in the United States (n = 36), followed by Australia (n = 25), India (n = 10), Canada (n = 5), the UK (n = 4), New Zealand (n = 3), Sweden (n = 2), Tanzania (n = 2), Finland (n = 1), Indonesia (n = 1), and Ireland (n = 1). Among the 56 articles reporting empirical evaluative data, 21 reported data collected using mixed-methods, 20 using quantitative methods, 11 qualitative methods, and 5 were literature syntheses. The vast majority of reported evaluative data were purely descriptive (e.g., the number of program participants; n = 34). Of studies attempting to measure efficacy, non-experimental before-and-after design was the most common method (n = 15), followed by retrospective self-report (n = 6), and quasi-experimental design (n = 4). Only two articles reported purely experimental (cluster-randomized) evaluation schemes. Interventions with mental health literacy components were the most frequently addressed (n = 46), followed by peer and paraprofessional support (n = 26), and direct clinical intervention (n = 19). Other prominent intervention components included financial or material aid, or both (n = 11), service network development (n = 10), physical health promotion (n = 7), and agroecological education (n = 6) (Figure 3).

### 3.2. Mental Health and/or Crisis Literacy

Mental health and crisis literacy programs were the most common and most evaluated components of mental health literacy interventions (n = 46). A total of fourteen articles addressed solely mental health or crisis literacy interventions, or both. A total of eleven of these provided evaluative data. A total of six studies evaluated mental health literacy interventions, which utilized Mental Health First Aid (MHFA), a country-specific standardized training course in which participants learn to understand the signs and symptoms associated with behavioral health issues and how to respond [67]. The earliest published article on MHFA describes a 12-h in-person workshop conducted as part of the New South Wales Farmers Blueprint for Mental Health in Australia, targeted “workers in agriculture-related roles who provide front-line financial, farm management and related assistance” [68]. Pre- and post-intervention surveys revealed that participants’ knowledge of mental health disorders and evidence-based interventions, as well as their confidence in their ability to provide help, increased significantly. A total of two later articles addressed 12-h MHFA workshops conducted in South Queensland, Australia, which more narrowly targeted Farm Advisors Employees of the Department of Primary Industries and Fisheries and the Department of Natural Resources and Water [69] and Advisory and Extension Agents [70]. Pre- and post-intervention surveys revealed a significant increase in mental health literacy and increased confidence in helping those with mental illnesses. In the US, Mississippi Extension agents who received an 8-h MHFA training retrospectively reported increased confidence to recognize mental disorders, utilize therapeutic communication skills, and assist a person in crisis [71]. A total of fifteen percent of trainees reported actually utilizing these skills with farmers in the 6 months post-training. Before-and-after evaluation of a Michigan Extension sponsored 8-h MHFA training “focusing on rural communities” also demonstrated that participants’ knowledge of mental health disorders and evidence-based interventions, as well as their confidence in their ability to provide help, increased [72].

Evaluative data from non-MHFA literacy programs also suggested effectiveness in increasing knowledge and confidence in helping behaviors. The in-person GoodYarn program in New Zealand is a brief (2.5 h), skill-based mental health literacy program developed for rural populations and delivered by paraprofessionals with agricultural backgrounds [73]. Retrospective surveys by GoodYarn participants reported increases in awareness, confidence, and knowledge about mental illness. The SCARF (Suspect, Connect, Ask, Refer, Follow-Up) program [74] held in New South Wales as part of the Farm-Link initiative is likewise briefer than MHFA (4 h) and tailored to an agricultural population. Frontline agricultural professionals who had received SCARF training primarily in workplace groups reported that they experienced significant increases in well-being, suicide literacy, and confidence to assist others in before-and-after surveys.

Pre- and post-survey data from the 4-h In the Know intervention for Canadian farmers [75] indicated significantly improved mental health knowledge and confidence in recognizing and intervening to aid farmers in distress. Pre- and post-survey data from the Farm Stress Training (FST) program for Farm Service Agents in the United States, a primarily self-paced online educational program with a 1-day in-person practice session, similarly indicated improved knowledge and skills in identifying and helping distressed farmers [76]. The 2- to 4-h mental health literacy program Communicating with Farmers Under Stress (CFS) was developed by Michigan State University Extension in the US to help agribusiness professionals understand and aid the mental health needs of the farming community [72]. In post-intervention retrospective questionnaires, the overwhelming majority of respondents reported increased knowledge of mental health and community-based mental health resources. In a follow-up survey completed 9–21 months post-training, nearly 90% of respondents reported increased comfort in talking with and helping those in distress, understanding of stress issues and warning signs of stress challenges, and knowledge of community resources. In addition, nearly 80% of respondents had shared workshop information and resources with others. Open-ended feedback suggested participants appreciated the training.

Ripple Effect, an online intervention designed to reduce stigma among males in the Australian farming community with a lived experience of suicide, represents the greatest departure from the MHFA format in that it was designed solely to reduce suicide stigma among farmers [77,78]. Pre- and post-survey data failed to show any significant reduction in self- or perceived-stigma [78]. However, qualitative data and participant feedback suggested stigma reduction.

Personal Expressions was an arts-oriented mental health promotion program designed to improve the self-esteem and self-efficacy of young people (ages 10–25) who were involved in youth farming programs in rural England as well as inform the public of the stressors faced by farmers [79]. Participants helped to determine the program design and received training and support from professional photographers as they used photography to share with the public the identities, work, challenges, and aspirations of young farmers. Qualitative feedback suggested that the intervention was successful in fostering self-confidence and esteem among young people with farming backgrounds.

Mental health and crisis literacy, particularly stress-management workshops, were described as key components of the vast majority of multi-component interventions deployed in the United States and Canada during the Farm Crisis and other times of crisis [37,55,80,81,82,83,84,85,86,87,88,89,90,91,92,93,94,95]. A total of three articles focused solely on mental health, crisis literacy, or both [59,88,96]. Unfortunately, limited evaluation data were collected, although anecdotal feedback from farmers suggests understanding the social, economic, and political context of the Farm Crisis to be therapeutic [84].

#### 3.2.1. Mental and Physical Health Literacy Promotion

A total of four articles addressed interventions which promoted both mental and physical health literacy; two targeted farming families and one targeted individual farmers [97,98,99,100]. Only one reported empirical evaluative data using a pre- and post-intervention mixed-methods design [97].

A total of two of these articles addressed Sustainable Farm Families of Australia, a 3-year long health-promotion program providing adult farming family members health assessments, provider referral, and health and wellness education workshops that included stress-management. In post-program quantitative surveys, participants’ knowledge related to health and welfare increased significantly and was retained over a 3-year period [97]. In addition, 24% of participants reported the program had motivated them to improve their well-being by increasing leisure and family time. Comments from focus groups held as part of the intervention also indicated that participants found the stress-management information helpful. Depression index and alcohol use behaviors were also collected pre-intervention, but any change post-intervention was unreported [98].

Although unevaluated, the Rural Adversity Mental Health Program (RAMHP) provided MHFA to over 3000 members of farming communities along with partnering with established services to promote mental and physical health [99]. Similarly, Farmstrong, a New Zealand-wide rural wellbeing initiative providing farmers practical, proactive measures on wellbeing, was described but not evaluated [100].

#### 3.2.2. Mental Health Literacy and Crisis Training for Healthcare Providers

As mentioned in the introduction, the shortage of mental health care providers in rural areas means that it is often non-mental health specialists such as general practitioners who eventually serve farmers with mental health conditions [54]. The lack of health providers who understand farming life and are well-versed in issues affecting the farming community is another major barrier to help-seeking [57]. Mental health literacy and crisis training for healthcare providers aims to address these issues. A total of six articles addressed mental health literacy initiatives for healthcare providers, two of which reported evaluative data. The Sustainable Farm Families Train the Trainer intervention in Victoria, Australia, trained healthcare providers on the Sustainable Farm Families program using a 5-day farming culture and crisis literacy workshop [101]. Focus groups held during the program suggested participants recognized the importance of the program. In addition, and also in Victoria, Deakin University developed an agricultural health and medicine postgraduate unit for mental health providers. In a survey of graduates, 73% reported using what they learned on mental health in the farming community in their current practice [102].

A total of six articles described but did not report evaluative data on interventions which trained existing healthcare providers and providers-in-training in farming and mental health literacy [37,55,93,99,103,104].

There are limited data available regarding the utility of mental health literacy programs for mental health prevention among farmer populations. Although popular programs such as MHFA have a broader research base, evidence of their use and effectiveness for farmer populations, or for those who work with farmers, relies on non-experimental self-reported data and is very limited. Additionally, several agencies have created their own training programs tailored specifically for agricultural populations; however, an evidence-base does not yet exist to provide strong support for their use. Overall, literacy programs appear to have value, but the lack of consistency across programs and the lack of rigorous evaluation are troubling.

### 3.3. Peer and Paraprofessional Support

A total of twenty-six articles addressed interventions with peer and paraprofessional support components. A total of seventeen addressed peer support interventions; sixteen addressed volunteer or paraprofessional interventions, or both. A total of fourteen addressed interventions in the US, seven in Australia, two in Canada, two in India, two in New Zealand, and one in the UK. A total of thirteen provided evaluative data.

#### 3.3.1. Support Groups and “Peer Listening”

A total of fourteen articles addressed purely peer support interventions. Only four reported evaluative data, and the data reported are solely qualitative: two in Australia [105,106], one in the UK [107], and one in New Zealand [73].

In Australia, farmers reported feeling immense relief from the “healing power of being able to talk to someone about their problems” and participating in informal support groups such as taking regular tea breaks at a local mechanic shop [105,106]. They believed these informal support groups could be effective in preventing rural male suicide. Industry association gatherings and activities were also identified as important sources of emotional support [105].

During the 2001 foot and mouth disease (FMD) crisis in the UK, non-profit Pentalk provided IT training, advice, and support to affected farmers, which allowed them to give and receive peer support during the lockdown [107]. Farmers reported that their access to the online community of local and global farmers affected by FMD was highly effective at reducing stress.

The New Zealand mental health literacy program GoodYarn (discussed under Mental Health and Crisis Literacy Interventions) employed a peer delivery model “where the workshops are delivered by people with both a farming or rural industry background and a personal or close personal experience of mental illness” [73]. In post-evaluation open-ended feedback surveys, participants expressed gratitude for the peer facilitator model and a preference for workshop facilitators to have farming backgrounds.

During the American Farm Crisis, Cooperative Extension, health providers, other institutions, and farmers themselves formed support groups for distressed farming families [37,55,60,80,83,84,85,92,95,108,109]. A total of two articles mention clinician facilitated support groups [110,111]. Also common were one-on-one “peer listening” and peer counseling programs [55,80,86,91,95,108]. Unfortunately, none of these articles provided evaluative data, but high rates of participation and anecdotal evidence suggest they were well-received by farmers [55,80] Moreover, in the landmark study on mental health of farm families during the Farm Crisis, respondents indicated that the most helpful form of aid was “moral and emotional support” [9.

A total of two articles [87,111] described, but did not report, evaluative data on post-Farm Crisis peer support interventions in the US.

#### 3.3.2. Paraprofessionally-Staffed Crisis Hotlines

A total of twelve articles described paraprofessionally-staffed crisis hotlines. Only one article described an entirely volunteer-staffed farm distress hotline [86] and one article described a hotline primarily staffed by volunteers, with clinical social workers providing “back-up” until clients were linked with community-centered behavioral services [90]. A total of one article described a crisis hotline staffed by hired paraprofessionals, the majority of whom were former farmers [112], and six additional articles described a crisis line but failed to specify who was staffing it [37,60,93,109,113,114]. A total of one article described a “career information hotline” where empathetic paraprofessional staff would help refer callers to resources including metal health services, but explicitly did “not provide counseling services” [115]. Of these 12 articles, only 3 provided descriptive data. The crisis hotlines of the multi-state initiative Sowing Seeds of Hope (SSoH) in the United States fielded more than 34,000 calls from 1999 to 2005, primarily from farm residents with concerns about family members, finances, and behavioral health issues [93]. The Extension-operated Rural Concern Hotline in Iowa fielded nearly 47,000 calls from 1985 to 1991 [112], and although the ratio of calls regarding financial issues declined in the late 80s, the ratio of callers who exhibited moderate to severe levels of stress has remained high. The career information hotline fielded 365 calls in the first 6 months from near equal proportions of farming men and women primarily “wanting to make a major career change” [115].

#### 3.3.3. Accidental Counselors

A total of twelve studies addressed the important counseling role of local professionals not trained in mental health. A total of eight reported evaluative data, including four studies which provided evidence of the role of Rural Financial Counsellors (RFCs) in Australia as important first responders for farmers in crisis as well as trusted sources of support [116,117,118,119]. In qualitative interviews of farmers by Alston [116], RFCs were described as “a culturally appropriate source of support and advice and lacking stigma” despite the fact that they were not trained to provide emotional counseling. In a quantitative survey of RFCs [117], Fuller and Broadbent found that nearly half (49%) of RFCs nationwide were referring a significant proportion of their clientele to social, emotional, and stress-related services. Fuller et al.’s network analysis of mental health related links between agricultural support, health, and other human services in rural New South Wales [118]; RFC was found to be the most highly linked service. Yet nearly half of RFCs (49%) reported that referrals were difficult because of clients’ unwillingness to acknowledge mental health problems or seek services, as well as the dearth of such services in rural areas [117].

Perceval and colleagues [106] describe how professional community members support farmers as “accidental counsellors.” Focus groups with dairy farmers in New Zealand reported similar findings with regards to rural veterinarians [120]. Unfortunately, interviews with rural medical professionals revealed that many of these non-professional counsellors suffer vicarious trauma from performing the emotional labor of counselling distressed farmers [121].

None of the articles addressing “accidental” counseling in the United States reported evaluative data. Cooperative Extension Agents were described as frequently providing instrumental as well as emotional counseling to distressed farmers [86,91,122]. A total of three articles described counseling interventions which focused exclusively on financial, vocational, or business advice, or a combination thereof [85,89,108]. Two articles described counseling interventions but did not indicate whether the counseling was provided by a clinically trained healthcare provider or a paraprofessional [60,93].

#### 3.3.4. Other Paraprofessional and Volunteer Interventions

The Rural Families Empowerment Program in the US was described as “one-on-one outreach education to farm and rural families facing severe stress,” combining proactive outreach, mental health education, along with case management to aid Iowa farm families in crisis [123]. Outreach counselors were “certified Family Development Specialists trained in group facilitation, dispute resolution, community development, diagnosis of mental health disorders, and crisis management.” In post-intervention surveys, participants reported improvement on 17 indicators of family well-being and indicated that the program was most helpful in solving debt and substance use problems, and managing financial problems. In Australia, paraprofessional drought support workers were deployed to drought-devastated communities, but their effectiveness was limited by their extremely short contract periods [116]. Australian farmers and fishermen reported that community volunteerism itself had a salutatory effect on mental health in addition to strengthening community social capital [105].

A total of five articles discussed paraprofessional “case-finder” program components, but none reported evaluative data; three addressed interventions in the US [37,87,93], and two addressed interventions in India [124,125].

Overall, there are limited evaluation data available related to peer and paraprofessional support related to farmer mental health. However, multiple methods of support have been provided. What little data are available suggest that these programs are well-received, and that social support is likely a valuable contribution to any attempts to provide mental health support to farmers.

### 3.4. Direct Clinical Intervention

A total of nineteen articles described interventions by clinical professionals, nine in the US, four in Australia, three in India, two in Canada, and two in the UK. Only two provided evaluative data.

#### 3.4.1. Crisis Counseling

A total of seventeen articles describe crisis counseling interventions by professional mental health clinicians [37,55,57,81,82,83,84,87,93,95,104,110,111,116,126,127,128]. Unfortunately, the evaluation data on these initiatives are limited and purely descriptive. The Farm Partners (FP) Program administered by the New York Center for Agricultural Medicine and Health in the US is described as a proactive social case work program whereby social workers made unscheduled visits to farmers referred to them by concerned family or community members and provide a wide range of counseling and referral services [87]. From 1992 to 1998, the program served 395 farmers and made over 700 referrals. Survey responses on client satisfaction rated the service 3.3 on a 4-point scale.

In Australia, RAMHP hired professional community mental health workers and responded to over 270 calls to its crisis hotline from 2007 to 2008 [99]. The Australian New South Wales Rural Mental Health Support Line, staffed by “mental health professionals,” reported an average of 48 callers per month, with even numbers of male and female callers who were primarily farming family members, support providers, and non-farmer community members [126]. Hotline staff reported that they believed the service to be helpful to farmers.

A total of two studies described but did not evaluate the deployment of mental health professionals offering broad-based psychosocial services to farmers in Australia [116] and Canada [57]. The author of the article describing the Canadian intervention cited a French language report suggesting “positive results notably on destigmatizing help-seeking and providing a proactive outreach to those people less likely to engage with professional services” [57].

None of the other articles addressing clinical counseling interventions in the US [37,55,81,82,83,84,93,95,104,110,111,127] or India [128] reported any evaluative data.

#### 3.4.2. Other Direct Clinical Interventions

A total of two articles described but did not report evaluative data on nurse-led health services developed for farmers in the UK [129,130]. A total of two articles addressed the introduction of additional psychiatrists to address farmer suicide in India [124,131]. Badami [131] contends that the poorly administered, purely psychiatric mental health policies and clinical services of the District Mental Health Programme (DMHP) in Kerala, India, was ill-equipped to deal with the complex reality of agrarian suicide.

It should also be noted that 23 articles addressed interventions which linked farm residents to community-based services including mental health professionals. However, only one article reported evaluative data; Rosmann [93] reported that SSoH linked more than 400 farm residents to healthcare providers since 1999.

There appears to be a presumption that direct clinical services are beneficial for participants, with limited evaluation available. However, it is notable that concerns have arisen related to some of the clinical services described in these articles. Deploying therapists for short contract periods appears to be problematic for continuity of care. Further, some of the psychiatric services provided in India indicated that there may need to be better preparation for providers and a more comprehensive approach to issues of farmer mental health.

### 3.5. Other Interventions

#### 3.5.1. Financial and Material Aid

A total of ten articles addressed interventions which provided direct financial or material aid, or both, to farmers, their farming families, or a combination of both. A total of five were located in the US, four in India, and one in Australia. Of the six articles which included evaluative components, evaluation was based on literature synthesis (n = 4), semi-structured interviews (n = 1), self-report (n = 1), and ethnography (n = 1).

In the US, farming families who had received financial aid in Wisconsin overwhelmingly reported that the non-profit which provided the aid was the most helpful resource in alleviating their troubles [114]. A total of four other material aid programs [81,91,93,108] were briefly described.

A total of three studies utilized literature synthesis [131,132,133] and one literature synthesis and ethnography [134] to evaluate debt-relief schemes in India. A government payment scheme in Kerala, India, to compensate the families of farmers who died by suicide due to “agrarian distress,” was critically evaluated and found to be ineffective and potentially harmful [131,132,134]. This scheme was a target of fraud and a bureaucratic nightmare to access. In addition, its funding was unsustainable, and it was criticized by mental health professionals as “encouraging people in debt to commit suicide” [131]. The second, a moratorium on farm loans, is described in slightly more positive tones [131,132,133], but all four authors suggest that these interventions are a facile, less costly appeasement to political pressure rather than an implementation of necessary but difficult structural solutions. In Australia, the immense bureaucratic difficulties and frustration in accessing Exceptional Circumstance (EC) payments was described based on interviews with farmers and farm community members in New South Wales, Australia [116].

#### 3.5.2. Service Network Development

A total of nine articles described interventions aiming to increase communication and collaboration among stakeholders providing services to farmers in distress such as farm advocacy organizations, healthcare providers, and rural advisors [68,83,91,93,99,111,135,136,137]. Only one of these articles reported evaluative data. Quantitative evaluation of Farm-Link, which delivered MHFA workshops and facilitated meetings between “workers from the health and welfare sector and agricultural support and service sectors,” indicated increased links between mental health-related services and other services in the target communities after the initiative [135]. Qualitative data were also taken in the form of interviews with three project staff who attributed much of the program’s success to its ability to engage the agricultural and health sectors together.

#### 3.5.3. Agroecological Education

A total of six articles addressed agroecological education initiatives with the aim of restoring both farmer mental health and local ecology by teaching farmers chemical input-free, environmentally sustainable polyculture. Non-Pesticide Management (NPM) was developed in India as an alternative to chemical-dependent farming in order to reduce financial distress and suicide caused by dependence on harmful chemical inputs [138]. Compared to the number of suicides in similar villages which had not adopted NPM, suicides in the pesticide-free communities decreased post-intervention. However, this difference was not significant due to the small sample size. The ideologically similar Zero-Budget Natural Farming (ZBNF) was promoted explicitly as a way to reduce indebtedness and suicide among Indian farmers [139]. Efficacy of ZBNF among farmers throughout Karnataka, was evaluated in a before-after study design using oral histories, semi-structured interviews, agricultural field visits, focus groups, participant-observation, and questionnaires. Farmers who converted to ZBNF from chemically-dependent farming reported significantly reduced stress, a more hopeful outlook, a stronger sense of purpose, and overall improved mental health.

A total of two articles addressed the Singida Nutrition and Agroecology Project, which taught agroecology methods designed to increase food diversity and food security among smallholder families in Tanzania through increasing biodiversity and increasing the cultivation of legumes [140,141]. A cluster-randomized efficacy trial demonstrated that levels of probable depression, as measured by the Center for Epidemiologic Studies Depression Scale (CES-D), decreased by 43% among women farmers in the intervention group [140]. Structural equation modelling revealed that the increase in house food security explained 23% of this impact [141].

Kākoʻo ʻŌiwi [142] was described as a non-profit initiative to restore human and cultural relationships to revitalized lo‘i kalo (traditional wetland and flooded field system agriculture) in Hawaii, US. Kākoʻo ʻŌiwi’s first goal was “to build capacity of farmers and provide local families with the opportunity to learn to farm and care for lo‘i kalo and (re)connect to the land” [142]. Efficacy of the program was measured qualitatively using participatory methods based on an indigenous cultural ecosystem service process and framework. The farming families participating in the program reported significantly increased “Ola Mau,” a Hawaiian term denoting mental and physical wellbeing, as a result of program participation. Improved mental wellbeing was described in terms of mental healing, self-acceptance, supportive social networks, and a sense of cultural and spiritual fulfillment.

Authors of a qualitive meta-analysis of literature on natural resource management (NRM) programs in Australia proposed that agroecological programs have the potential to be deployed as non-traditional mental health interventions [143]. However, this hypothesis has yet to be empirically tested.

#### 3.5.4. Political Activism

A total of five articles suggested that participation in pro-farmer political action groups advocating humane farm policy may reduce stress and alleviate mental health problems in this population [57,89,92,122,134]. In his ethnographic analysis on farmer suicide in Kerala, India, Münster [134] noted that compared with the general farmer population, farmers participating in social movements rarely committed suicide.

#### 3.5.5. Advocacy

A total of four articles described but did not evaluate the role of organizations advocating for increased attention to agricultural mental health; two addressed interventions in Canada [57,84], one an intervention in Australia [105], and one an intervention in the US [93].

#### 3.5.6. Physical Health Promotion

A total of three articles addressed physical health promotion programs: one in Australia, one in Ireland, and one in the US. All three reported evaluative data, one using pre- and post-intervention design, one used a quasi-experimental design, and one was descriptive. All three targeted individual farmers.

The earliest intervention, two farm health fairs held in the midwestern United States, invited local farmers to a free health screening and education event. It then mailed them individualized health recommendations after the event based on their health histories and health screening results taken at the fair [144]. A total of nine percent of respondents in a follow-up telephone survey reported strengthening stress reduction support systems because of the fair.

Farming Fit in Australia [145] and a farmers fitness program in Ireland [146] took more proactive approaches. In Farming Fit, overweight and obese farmers were assigned individualized exercise programs over a 6-month period. An exercise physiologist and research assistant would monitor participant progress and be available for support. Researchers measured no difference between the intervention and control group for overall mental health at follow up, or a difference in cortisol levels. Rather than an individualized plan, a farmers fitness program in Ireland [146] held 2-h group exercise training sessions and 1-h lifestyle education trainings for obese and overweight farmers once a week over a period of 6 weeks. Mental health scores of participants significantly improved post-intervention.

#### 3.5.7. Research Initiatives

A total of three articles described research interventions. Rosmann [37] argued that the establishment of agromedicine research and training programs with behavioral health components such as the National Farm Medicine Center and journals such as the Journal of Agricultural Safety and Health, university-based programs, and non-university-based agricultural safety and health programs and organizations such as the AgriSafe Network are important players in addressing issues in the farming population despite the chronic underfunding of behavioral health components. Human and Wasem [104] also asserted that increased research funding on farmer mental health led to effective interventions. Roy [57] described the therapeutic effect of his qualitative research process itself on interviewee farmers.

#### 3.5.8. Community-Based Participatory Research

A total of two studies utilized community-based participatory research to design interventions, including farmers and community members as full and equal partners in designing and evaluating interventions. Both studies reported evaluative data. A suicide prevention education and training program in India utilized a community-based participatory research design to involve farmers, community leaders, organizational representatives, farmers educators, students, healthcare providers, and pesticide shop owners in developing educational material and training community stakeholders [147]. The educational materials were designed to educate various populations within the community on mental and behavioral health literacy, deliberate self-harm (DSH) and suicide prevention, violence against women, and safe pesticide use. Evaluation was based on feedback from target groups and pre- and post-intervention number of admissions to the local public health center for DSH and suicide. Feedback from target groups rated the educational materials as highly useful, and admissions to the local public health center for DSH and suicide declined post-intervention.

Researchers in Indonesia recruited farmers into focus groups in order to promote recognition of health issues of farmers, analyze those issues, and develop solutions in coordination with public health centers [148]. The schema developed included community-level health education, social support groups, and health support workers. Qualitative feedback from focus groups indicated farmers were taking steps to better manage stress and improve their overall health.

#### 3.5.9. Occupational Safety Programs

A total of two articles evaluated the impact of an occupational safety training program in Sweden [149,150]. Pre-, post-, and follow-up questionnaires of participants in an intervention group and control group showed that work stress decreased immediately post-intervention and effects remained at follow-up. However, the control group also showed a decline in work stress, indicating that stress reduction may have had other causes than the intervention [149].

#### 3.5.10. Technological Innovation

A total of one article evaluated a technological innovation which reduced occupational stress in Finnish farmers. In an evaluation of farmers who had adopted automatic milking systems (AMS) for dairy cows, 47.8% reported that adopting AMS reduced their mental stress, 19.7% reported no difference in stress after adopting AMS, and 31.6% stated that their mental stress had increased [151].

#### 3.5.11. Skill Training Programs

A total of six articles briefly mentioned skill training programs designed to improve financial well-being and family functioning, but did not report evaluative data [60,83,85,90,91,92].

#### 3.5.12. Farmer Cooperativization

A total of one article briefly described the development of cooperatives as part of a mental health intervention during the American Farm Crisis [55]. No evaluative data were reported on this intervention.

#### 3.5.13. Recommendations for Intervention Programming

Although not describing specific interventions, four articles provided evidence-based recommendations for future mental health interventions in farming populations; three addressed farming populations in the US [61,94,152] and one addressed farmers in Australia [153].

## 4. Discussion

Around the globe, community leaders and farmers themselves have galvanized resources to address stress, mental health issues, and suicide among farmers. Although evidence suggests that many of these programs proved helpful, unfortunately, rigorous evaluation of most of these interventions was severely lacking. Most English-language published articles provided only descriptive evidence, and those that did provide evaluative data overwhelmingly relied on self-report using non-standardized measures. Much of the literature addressed multiple interventions or multi-component interventions, proscribing evaluation of specific mechanisms. Only one intervention was of the gold-standard experimental design, and its narrow target of food-insecure female farmers with young children in Tanzania limits generalizability to other farming populations [140,141]. The most consistent methodology used was self-report questionnaires concerning mental health literacy programs. Data from these questionnaires suggested that these programs significantly improve knowledge, attitudes, and helping behaviors among workers in farmer-facing professions [68,70,75,97]. However, this evaluation was based entirely on self-report and does not reflect the experience of the farmers who are ostensibly the recipients of help. Only two mental health literacy intervention evaluations attempted to measure mental health improvements among actual farmers [74,79] and none reported long-term impact. In addition, in 6-month follow-up evaluations, a substantial portion of trainees report that their limited contact time with farmers and farmers’ unwillingness to talk about mental health issues impede their ability to actually utilize the skills [71]. Several researchers on farmer suicide in Australia have noted that although mental health literacy campaigns in general are effective in raising awareness of mental health issues, there is little evidence yet that they have led to changes in farmers’ behaviors or increased their willingness to seek help, and they have not resulted in fewer suicides [106,154,155]. Stigma directed at help-seeking and mental health issues remains endemic despite these efforts [106].

Peer and paraprofessional support interventions were similarly understudied. Although qualitative accounts and retrospective surveys reported farmers benefiting from the emotional support they received from peers and paraprofessionals, there is evidence that taking on the role of “accidental counselor” can in turn harm the mental health of those helping [121]. Outside of client satisfaction surveys, evidence for direct clinical interventions was nonexistent. Qualitative studies suggested significant promise in agroecological interventions and community-based participatory research, but more robust evaluation in a variety of farming contexts utilizing consistent methodology is needed. Evidence for efficacy of other interventions was weak, mixed, or both.

The dearth of evaluation of holistic, multi-component programs is the most unfortunate, however, as a large body of evidence in mental health interventions suggests that multilevel initiatives increase the odds of effectively countering stress, treating mental illness, and preventing suicide [92,156,157,158]. Multi-component interventions included in this review such as Sowing Seeds of Hope (SSoH) [93] and the Rural Adversity Mental Health Program (RAMHP) [99], although lacking robust evaluation, attracted high numbers of service users. The evidence is clear that farmers in distress often require a variety of resources: emotional counseling, an understanding of mental illness, technical advice, financial consultation, legal advice, social support, emergency needs, and vocational counseling, etc. Indeed, the overall lack of integrated human service delivery was the most criticized aspect of mental health interventions by both researchers and the farmers they interviewed [92,104,116,131]. The difficulties in determining efficacy of concurrent components appears to have led to an emphasis on single interventions whose impacts are empirically and unambiguously measurable, despite the fact that holistic approaches are theoretically the most effective.

### 4.1. Limitations

This review has several limitations. First, our search terms may not have captured all interventions published in peer-reviewed journals. Given a broader search, we might have included papers that utilized differing terminology in their interventions. Second, we have only included results published in English, which, while providing an international perceptive, restricted the focus to mainly high-income, Anglophone countries. The dearth of research from low-income countries may be due to publishing language barriers, limited resources for research, or both, rather than a dearth of interventions. Third, this review only examined interventions described in published literature. There are likely interventions devised and used that remain unpublished. Fourth, only two authors were involved in the study selection; as there was full consensus between the authors on study inclusion, no third evaluator was consulted. Finally, as the international systematic review database (PROSPERO) was not accepting registrations for literature reviews at the time the study protocol was completed, there was no formal registration of the study protocol.

### 4.2. Directions for Further Research

There is an urgent need for a stronger and broader evidence base in the field of mental health interventions among farmers. Perhaps most imperative is the development of more effective evaluation methodologies for multi-component interventions, which theoretically suggest the most promise but are the least evaluated. For more focused interventions such as mental health literacy, there is a need for efficacy measures to focus more directly on indicators of farmer well-being as well as long-term effects. As qualitative research suggests that interventions which include farmers’ views are acceptable and effective, more research needs to engage farmers in developing interventions which addresses specific social, environmental, and cultural factors affecting mental health.

## 5. Conclusions

Our review has demonstrated that despite the high number and wide variety of farmer mental health interventions implemented globally in the past 50 years, very few received robust evaluation. This is especially true of the multitude of programs implemented during and after the American Farm Crisis. Evaluation of mental health literacy programs shows the most consistency but lacks construct validity. Evaluation of agroecological and community-based participatory research interventions appear to have high levels of validity but lack reliability. Although many of the programs reported high levels of usage by farmers, the unfortunate dearth of evaluation and inconsistency across interventions proscribes empirically-based conclusions about differing degrees of effectiveness.

## Appendix A

**Table A1:** Population-Intervention-Comparison-Outcome-Study Design (PICOS).

	Description	Inclusion/Exclusion Criteria
Population	Primary agricultural producers, their families, members of farming/agricultural communities, or community stakeholders.	The study sample or a substantial number of subjects (minimal 50%) are represented in the study population. Studies conducted in agricultural-oriented countries or communities where farmers or farm family members were not explicit targets were excluded.
Intervention	Reducing psychological distress, promoting mental health, improving mental health outcomes and/or reducing suicide among primary agricultural producers.	Purely descriptive studies, as well as those that measured intervention efficacy were included. Studies of interventions with the aim of improving the mental health of solely farmworkers (not producers), studies of substance use disorder interventions, and studies of suicide means restriction were excluded.
Comparison	No control group or comparison is required.	
Outcome	No measurable outcome is required.	
Study design	Peer-reviewed studies are included.	No restrictions on the type of studies, geographic region, or publication dates.
		Articles published in languages other than English were excluded.

## Appendix B

**Table A2:** Table with Details of Included Articles.

Ref ID	Author	Year	Title	Journal	A	B	C	D
[102]	Adams, J., Cotton, J., and Brumby, S.	2020	Agricultural health and medicine education: Engaging rural professionals to make a difference to farmers’ lives	Australian Journal of Rural Health	✖			
[55]	Adams, R. D., and Benjamin, M. L.	1988	Innovative approaches to mental health service delivery in rural areas	Journal of Rural Community Psychology	✖	✖	✖	✖
[116]	Alston, M.	2007	“It’s really not easy to get help”: Services to drought-affected families	Australian Social Work		✖	✖	✖
[124]	Alt, S.	2018	Farmer suicides and the function of death in neoliberal biopolitics	International Political Sociology			✖	
[131]	Badami, S.	2014	Suicide as a counter-narrative in Wayanad, southern India: The invisible death	South Asia Research			✖	✖
[98]	Blackburn, J., Brumby, S., Willder, S., and McKnight, R.	2009	Intervening to improve health indicators among Australian farm families	Journal of Agromedicine	✖			
[80]	Blundall, J	1986	The initial response from a community mental health center.	Human Services in the Rural Environment	✖	✖		
[81]	Bosch, K. R.	2004	Cooperative Extension responding to family needs in time of drought and water shortage	Journal of Extension	✖		✖	
[96]	Breeze, M., Crowder, L., Jones, D., and Taylor, M.	1987	Getting help: The first step towards farm health	Extension Review	✖			
[142]	Bremer, L. L., Burnett, K. M., Ching, C., Chun, G., Falinski, K., Kukea-Shultz, K., Oleson, K. L. L., Reppun, N., Ticktin, T., and Wada, C. A.	2018	Biocultural restoration of traditional agriculture: Cultural, environmental, and economic outcomes of Lo‘i Kalo restoration in He‘eia, O‘ahu	Sustainability				✖
[97]	Brumby, S. A., Willder, S. J., and Martin, J.	2009	The Sustainable Farm Families project: Changing attitudes to health	Rural Remote Health	✖			
[101]	Brumby, S., and Smith, A.	2009	‘Train the trainer’ model: Implications for health professionals and farm family health in Australia	Journal of Agromedicine	✖			
[145]	Brumby, S., Chandrasekara, A., Kremer, P., Torres, S., McCoombe, S., and Lewandowski, P.	2013	The effect of physical activity on psychological distress, cortisol and obesity: results of the Farming Fit intervention program	BMC Public Health				✖
[82]	Cecil, H. F.	1988	Stress: Country Style: Illinois response to farm stress	Journal of Rural Community Psychology	✖		✖	
[140]	Cetrone, H. M., Santoso, M. V., Bezner Kerr, R., Petito, L., Blacker, L., Nonga, T., Martin, H. D., Kassim, N., Mtinda, E., and Young, S. L.	2021	Food security mediates the decrease in women’s depressive symptoms in a participatory nutrition-sensitive agroecology intervention in rural Tanzania	Public Health Nutrition				✖
[147]	Chowdhury, A. N., Banerjee, S., Brahma, A., and Biswas, M. K.	2013	Participatory research for preventing pesticide-related DSH and suicide in Sundarban, India: A brief report	ISRN Psychiatry				✖
[83]	Crawford, C.	1986	Response to the rural crisis: Missouri Cooperative Extension Service.	Human Services in the Rural Environment	✖	✖	✖	✖
[126]	Crockett, J. A., Hart, L., and Greig, J.	2009	Assessment of the efficacy and performance of the New South Wales Rural Mental Health Support line	Australian Journal of Rural Health			✖	
[76]	Cuthbertson, C., Brennan, A., Shutske, J., Zierl, L., Bjornestad, A., Macy, K., Schallhorn, P., Shelle, G., Dellifield, J., Leatherman, J., Lin, E., and Skidmore, M.	2020	Developing and implementing Farm Stress Training to address agricultural producer mental health	Health Promotion	✖			
[72]	Cuthbertson, C., Eschbach, C., and Shelle, G.	2021	Addressing farm stress through Extension mental health literacy programs	Journal of Agromedicine	✖			
[108]	DeLind, L. B.	1986	The U.S. farm crisis: Program responses and alternatives to them: The case of Michigan	Agriculture and Human Values		✖		✖
[127]	Dickens, S., Dotter, E., Handy, M., and Waterman, L.	2014	Reducing stress to minimize injury: the nation’s first employee assistance program for dairy farmers	Journal of Agromedicine			✖	
[136]	Fragar, L., Kelly, B., Peters, M., Henderson, A., and Tonna, A.	2008	Partnerships to promote mental health of NSW farmers: The New South Wales Farmers Blueprint for Mental Health	Australian Journal of Rural Health				✖
[119]	Fuller, J. D., Kelly, B., Law, S., Pollard, G., and Fragar, L.	2009	Service network analysis for agricultural mental health	BMC Health Services Research		✖		
[117]	Fuller, J., and Broadbent, J.	2006	Mental health referral role of rural financial counsellors	Australian Journal of Rural Health		✖		
[118]	Fuller, J., Kelly, B., Sartore, G., Fragar, L., Tonna, A., Pollard, G., and Hazell, T.	2007	Use of social network analysis to describe service links for farmers’ mental health	Australian Journal of Rural Health		✖		
[84]	Gerrard, N.	2000	An application of a community psychology approach to dealing with farm stress	Canadian Journal of Community Mental Health	✖	✖	✖	✖
[85]	Gladwin, C. H.	1988	Innovative extension projects for part-time farmers: There is no funding	Choices	✖	✖		✖
[153]	Gunn, K. M., Barrett, A., Hughes-Barton, D., Turnbull, D., Short, C. E., Brumby, S., Skaczkowski, G., and Dollman, J.	2021	What farmers want from mental health and wellbeing-focused websites and online interventions	Journal of Rural Studies				✖
[107]	Hagar, C., and Haythornthwaite, C.	2005	Crisis, farming and community	Journal of Community Informatics		✖		
[75]	Hagen, B. N. M., Harper, S. L., O’Sullivan, T. L., and Jones-Bitton, A.	2020	Tailored mental health literacy training improves mental health knowledge and confidence among Canadian farmers	International Journal of Environmental Research and Public Health	✖			
[99]	Hart, C. R., Berry, H. L., and Tonna, A. M.	2011	Improving the mental health of rural New South Wales communities facing drought and other adversities	Australian Journal of Rural Health	✖		✖	✖
[115]	Heppner, M. J., Johnston, J. A., and Brinkhoff, J.	1988	Creating a Career Hotline for Rural Residents	Journal of Counseling & Development		✖		
[122]	Herrick, J. M.	1986	Farmers revolt! Contemporary farmers’ protests in historical perspective: Implications for social work practice	Human Services in the Rural Environment		✖		✖
[121]	Hossain, D., Eley, R., Coutts, J., and Gorman, D.	2008	Mental health of farmers in Southern Queensland: Issues and support	Australian Journal of Rural Health		✖		
[70]	Hossain, D., Gorman, D., and Eley, R.	2009	Enhancing the knowledge and skills of advisory and Extension agents in mental health issues of farmers	Australasian Psychiatry		✖		
[69]	Hossain, D., Gorman, D., Eley, R., and Coutts, J.	2009	Farm advisors’ reflections on Mental Health First Aid Training	Australian e-Journal for the Advancement of Mental Health		✖		
[86]	Hughes R., Jr.	1987	Empowering rural families and communities	Family Relations	✖	✖		
[129]	Hughes, H. W., and Keady, J.	1996	The Strategy for Action on Farmers’ Emotions (SAFE): working to address the mental health needs of the farming community	Journal of Psychiatric and Mental Health Nursing			✖	
[104]	Human, J., and Wasem, C.	1991	Rural mental health in America	American Psychologist	✖		✖	
[151]	Karttunen, J. P., Rautiainen, R. H., and Lunner-Kolstrup, C.	2016	Occupational health and safety of Finnish dairy farmers using automatic milking systems	Frontiers in Public Health				✖
[146]	Kavanagh, R., Cooper, D., Bolton, J., and Keaver, L.	2021	The impact of a 6-week community-based physical activity and health education intervention-a pilot study among Irish farmers	Irish Journal of Medical Science				✖
[59]	Keating, N. C.	1987	Reducing stress of farm men and women	Family Relations	✖			
[77]	Kennedy, A. J., Versace, V. L., and Brumby, S. A.	2016	Research protocol for a digital intervention to reduce stigma among males with a personal experience of suicide in the Australian farming community	BMC Public Health	✖			
[78]	Kennedy, A. J., Brumby, S. A., Versace, V. L., and Brumby-Rendell, T.	2020	The ripple effect: a digital intervention to re-duce suicide stigma among farming men	BMC Public Health	✖			
[152]	Ketterman, J., Braun, B., & Pippidis, M.	2020	Extension programming resource for building farm and farm family resilience	Journal of Extension				✖
[105]	Kilpatrick, S., Willis, K., Johns, S., and Peek, K.	2012	Supporting farmer and fisher health and wellbeing in ‘difficult times’: Communities of place and industry associations	Rural Society		✖		✖
[87]	May, J. J.	1998	The Farm Partners Program	Journal of Agromedicine	✖	✖	✖	
[88]	McMoran, D., Seymour, K., Bachtel, S., and Thorstenson, S.	2019	Creating a Suicide Prevention Program for Farmers and Farmworkers	Journal of Extension	✖			
[139]	Meek, D., and Khadse, A.	2020	Food sovereignty and farmer suicides: bridging political ecologies of health and education	Journal of Peasant Studies				✖
[89]	Mermelstein, J.	1986	Farm Counseling Services, Inc.	Human Services in the Rural Environment	✖	✖		
[90]	Mermelstein, J.	1986	MOFARMS	Human Services in the Rural Environment		✖		✖
[110]	Mermelstein, J., and Sundet, P.	1988	Factors influencing the decision to innovate: The future of community responsive programming	Journal of Rural Community Psychology		✖	✖	
[91]	Molgaard, V. K.	1997	The Extension service as key mechanism for research and services delivery for prevention of mental health disorders in rural areas	American Journal of Community Psychology	✖	✖		✖
[112]	Molgaard, V. K., and Phillips, F.	1991	Telephone hotline programming	Journal of Extension		✖		
[73]	Morgaine, K., Thompson, L., Jahnke, K., and Llewellyn, R.	2017	GoodYarn: Building mental health literacy in New Zealand’s rural workforce	Journal of Public Mental Health	✖	✖		
[134]	Münster, D.	2012	Farmers’ suicides and the state in India: Conceptual and ethnographic notes from Wayanad, Kerala	Contributions to Indian Sociology				✖
[128]	Nayak, R. B., Bhatia, T., Mahadevaiah, M., and Bheemappa, A.	2020	Effectiveness of Psychological Intervention by Videoconference for Family Members with Depression of Farmers Who Have Committed Suicide	Indian Journal of Psychological Medicine			✖	
[92]	Olson, K. R., and Schellenberg, R. P	1986	Farm stressors	American Journal of Community Psychology	✖	✖		✖
[135]	Perceval, M., Fuller, J., and Holley, A. M.	2011	Farm-link: Improving the mental health and well-being of people who live and work on NSW farms	International Journal of Mental Health				✖
[74]	Perceval, M., Reddy, P., Ross, V., Joiner, T., and Kolves, K.	2020	Evaluation of the SCARF well-being and suicide prevention program for rural Australian communities	Journal of Rural Health	✖			
[106]	Perceval, M., Ross, V., Kõlves, K., Reddy, P., and De Leo, D.	2018	Social factors and Australian farmer suicide: a qualitative study	BMC Public Health		✖		
[100]	Roberts, R., Lockett, H., Bagnall, C., Maylea, C., and Hopwood, M.	2018	Improving the physical health of people living with mental illness in Australia and New Zealand.	Australian Journal of Rural Health	✖			
[71]	Robertson, M. N., DeShong, H. L., Steen, J. K. S., Buys, D. R., and Nadorff, M. R.	2021	Mental health first aid training for Extension agents in rural communities	Suicide and Life-Threatening Behavior	✖			
[103]	Robinson, T., Kurtz, H., Kelly, B., Fuller, J., Fragar, L., Roy, S., Deans, K., Croft, J., and Hedger, B.	2009	Clinical leadership in rural psychiatry: Farmers’ mental health and well-being	Australian Journal of Rural Health	✖			
[93]	Rosmann, M. R.	2005	Sowing the seeds of hope: providing regional behavioral health supports to the agricultural population	Journal of Agricultural Safety and Health	✖	✖	✖	✖
[37]	Rosmann, M. R.	2008	Behavioral healthcare of the agricultural population: A brief history	Journal of Rural Mental Health	✖	✖	✖	
[57]	Roy, P., Duplessis-Brochu, E., and Tremblay, G.	2019	Responses to adversity faced by farming men: A gender-transformative analysis	International Journal of Child Youth & Family Studies			✖	✖
[94]	Rudolphi, J. M., Berg, R., and Marlenga, B.	2019	Who and how: Exploring the preferred senders and channels of mental health information for Wisconsin farmers	International Journal of Environmental Research and Public Health	✖			✖
[144]	Rydholm, L., and Kirkhorn, S. R.	2005	A study of the impact and efficacy of health fairs for farmers	Journal of Agricultural Safety and Health				✖
[141]	Santoso, M. V., Bezner Kerr, R. N., Kassim, N., Martin, H., Mtinda, E., Njau, P., Mtei, K., Hoddinott, J., and Young, S. L.	2021	A nutrition-sensitive agroecology intervention in rural Tanzania increases children’s dietary diversity and household food security but does not change child anthropometry: Results from a cluster-randomized trial	Journal of Nutrition				✖
[68]	Sartore, G.-M., Kelly, B., Stain, H. J., Fuller, J., Fragar, L., and Tonna, A.	2008	Improving mental health capacity in rural communities: Mental Health First Aid delivery in drought-affected rural New South Wales	The Australian Journal of Rural Health	✖			✖
[143]	Schirmer, J., Berry, H. L., and O’Brien, L. V.	2013	Healthier land, healthier farmers: considering the potential of natural resource management as a place-focused farmer health intervention	Health & Place				✖
[109]	Schulman, M. D., and Armstrong, P. S.	1990	Targeting farmers for stress reduction	Journal of Extension		✖		
[111]	Schweitzer, R. A., Deboy, G. R., Jones, P. J., and Field, W. E.	2011	AgrAbility mental/behavioral health for farm ranch families with disabilities	Journal of Agromedicine		✖	✖	✖
[113]	Slee, P.	1988	Crisis on the farm: A telephone survey of rural stress	Australian Social Work		✖		
[132]	Sridhar, V.	2006	Why do farmers commit suicide? The case of Andhra Pradesh	Economic and Political Weekly				✖
[120]	Stanley-Clarke, N.	2019	The role of agricultural professionals in identifying, mitigating and supporting farming families during times of stress: Findings of a qualitative study	Australian Journal of Rural Health		✖		
[149]	Stave, C., Pousette, A., and Torner, M.	2008	Risk and safety communication in small enterprises—how to support a lasting change towards work safety priority	Journal of Risk Research				✖
[150]	Stave, C., Torner, M., and Eklof, M.	2007	An intervention method for occupational safety in farming—evaluation of the effect and process	Applied Ergonomics				✖
[95]	Stevens, W.	1986	Rural Interventions in Southwest Minnesota: A Private Nonprofit Human Service Agency	Human Services in the Rural Environment	✖	✖	✖	
[148]	Susanto, T., Rahmawati, I., and Wantiyah.	2020	Community-based occupational health promotion programme: an initiative project for Indonesian agricultural farmers	Health Education				✖
[79]	Syson-Nibbs, L., Robinson, A., Cook, J., and King, I.	2009	Young farmers’ photographic mental health promotion programme: A case study	Arts & Health: An International Journal of Research, Policy and Practice	✖			
[133]	Taylor, M.	2011	‘Freedom from poverty is not for free’: Rural development and the microfinance crisis in Andhra Pradesh, India	Journal of Agrarian Change				✖
[60]	Thompson, E. A., and McCubbin, H. I.	1987	Farm families in crisis: An overview of resources	Family Relations		✖		✖
[137]	Tinc, P. J., and Sorensen, J. A.	2020	Stakeholders Team up for Action in New York Dairy (STAND): A collaborative action-planning workshop to combat toxic stress among New York dairy farmers	Journal of Agromedicine				✖
[125]	Travasso, C.	2015	Maharashtra government launches mental health programme to reduce suicide in farmers	British Medical Journal		✖		
[123]	Viegas, S., and Meek, J.	1998	The rural families program makes a difference	Journal of Extension		✖		
[138]	Vijayakumar, L., and Satheesh-Babu, R.	2009	Does ‘no pesticide’ reduce suicides?	International Journal of Social Psychiatry				✖
[130]	Walsh, M.	2000	A nurse practitioner-led farmers’ health service: setting up and evaluating a UK project	Australian Journal of Rural Health			✖	
[61]	Weigel, R. R.	1980	Helping farmers handle stress	Journal of Extension	✖			
[114]	Williams, R. T. (1996).	1996	The on-going farm crisis: Extension leadership in rural communities	Journal of Extension		✖		✖

Intervention Category Key: A: Mental Health and/or Crisis Literacy; B: Peer and Paraprofessional Support; C: Direct Clinical Intervention; D: Other Interventions.
